# Inhibitory Effect of Methyleugenol on IgE-Mediated Allergic Inflammation in RBL-2H3 Cells

**DOI:** 10.1155/2015/463530

**Published:** 2015-04-16

**Authors:** Feng Tang, Feilong Chen, Xiao Ling, Yao Huang, Xiaomei Zheng, Qingfa Tang, Xiaomei Tan

**Affiliations:** ^1^School of Traditional Chinese Medical Sciences, Southern Medical University, Guangzhou 510515, China; ^2^Guangdong Province Key Laboratory of Chinese Medicine Pharmaceutics, Southern Medical University, Guangzhou 510515, China

## Abstract

Allergic diseases, such as asthma and allergic rhinitis, are common. Therefore, the discovery of therapeutic drugs for these conditions is essential. Methyleugenol (ME) is a natural compound with antiallergic, antianaphylactic, antinociceptive, and anti-inflammatory effects. This study examined the antiallergic effect of ME on IgE-mediated inflammatory responses and its antiallergy mechanism in the mast cell line, RBL-2H3. We found that ME significantly inhibited the release of *β*-hexosaminidase, tumor necrosis factor- (TNF-) *α*, and interleukin- (IL-) 4, and was not cytotoxic at the tested concentrations (0–100 *μ*M). Additionally, ME markedly reduced the production of the proinflammatory lipid mediators prostaglandin E_2_ (PGE_2_), prostaglandin D_2_ (PGD_2_), leukotriene B_4_ (LTB_4_), and leukotriene C_4_ (LTC_4_). We further evaluated the effect of ME on the early stages of the Fc*ε*RI cascade. ME significantly inhibited Syk phosphorylation and expression but had no effect on Lyn. Furthermore, it suppressed ERK1/2, p38, and JNK phosphorylation, which is implicated in proinflammatory cytokine expression. ME also decreased cytosolic phospholipase A_2_ (cPLA_2_) and 5-lipoxygenase (5-LO) phosphorylation and cyclooxygenase-2 (COX-2) expression. These results suggest that ME inhibits allergic response by suppressing the activation of Syk, ERK1/2, p38, JNK, cPLA_2_, and 5-LO. Furthermore, the strong inhibition of COX-2 expression may also contribute to the antiallergic action of ME. Our study provides further information about the biological functions of ME.

## 1. Introduction

Allergic airway diseases, such as asthma and allergic rhinitis, are common diseases caused by hypersensitivity of the immune system. Approximately 10–20% of the world population is affected by allergies, with the number of allergy patients increasing annually [1, 2]. Most allergy patients are genetically predisposed to produce IgE. Mast cells are a key player in early allergic response, which typically occurs within minutes of exposure to an appropriate antigen, and other biological responses, including inflammatory disorders [3]. These cells are critical effector cells in IgE-dependent immediate hypersensitivity reactions [4]. Mast cell degranulation can initiate an acute inflammatory response and contribute to the progression of chronic diseases [5]. When an IgE-antigen binds with Fc*ε*RI, the receptor is activated, and a variety of biologically active mediators are released, causing allergic reactions, including the release of *β*-hexosaminidase, a common degranulation marker, histamine, arachidonic acid metabolites, and inflammatory cytokines [6]. Importantly, arachidonic acid metabolites, including prostaglandins and leukotrienes, mediate acute and chronic allergic reactions [7, 8]. RBL-2H3 cells are a mast cell line that originated from rat basophilic leukemia and have been widely used to study IgE-Fc*ε*RI interactions and degranulation. Furthermore, RBL-2H3 cells are a useful model for* in vitro* screening of antiallergy drug candidates.

The MAP kinase cascade is an important signaling pathway that regulates the differentiation, activation, proliferation, degranulation, and migration of immune cells, including mast cells [9]. MAPK signaling molecules are divided into three groups: extracellular signal-regulated kinase (ERK) 1/2, p38 MAPK, and c-JunNH2-terminal kinase (JNK) 1/2. Erk1/2 is an essential signal in the production of interleukin- (IL-) 5, tumor necrosis factor- (TNF-) *α*, IL-3, and IL-13 in mast cells [10]. p38 MAP kinase stimulates IL-4 production in bone marrow mast cells (BMMCs) [11]. Additionally, the activation of JNK is also responsible, at least partially, for the expression and production of several cytokines, including TNF-*α*, IL-2, and IL-6 in mast cells [12, 13].

Methyleugenol (ME,1-allyl-3,4-dimethoxybenzene) is an analog of the phenolic compound eugenol, and it is found in essential oils, including basil, anise, clove, lemon grass, and laurel leaf oils. In East Asia, ME is found in the essential oil fraction of* Asiasari radix* (Xixin in Chinese). It is used as a flavoring substance in dietary products, including cookies, ice cream, and nonalcoholic beverages, and is found in cosmetics, shampoos, soaps, fragrances, and herbal products in Europe, the USA, and other countries [14]. Previous work indicates that ME exerts antiallergic [15], antispasmodic [16], antinociceptive [14], and anti-inflammatory [17] effects. It was reported that ME inhibited passive cutaneous anaphylaxis (PCA) in rats, release of 5-lipoxygenase (5-LO) from RBL-1 cells and leukotriene D_4_ (LTD_4_) induced constriction of guinea pig ileum. ME also inhibited compound 48/80-induced systemic anaphylaxis and antidinitrophenyl IgE-induced local anaphylaxis in mice [18]. However, the effects of ME on allergic response in IgE-activated RBL-2H3 cells and its antiallergic mechanism remain unknown.

In this study, we investigated the antiallergic effects of ME in IgE-activated RBL-2H3 cells. Furthermore, we evaluated the mechanisms responsible for the antiallergic effects of ME.

## 2. Materials and Methods

### 2.1. Reagents

ME was purchased from the National Institute for Food and Drug Control (Beijing, China; purity, ≥99.5%). Dulbecco's minimum essential medium (DMEM), penicillin, streptomycin, and fetal bovine serum (FBS) were purchased from GIBCO (Grand Island, NY, USA). 4-[3-(4-Iodophenyl)-2-4(4-nitrophenyl)-2H-5-tetrazolio]-1,3-benzene disulfonate (WST-1) was obtained from Dojindo (Kumamoto, Japan). Specific antibodies against phospho-Lyn, Lyn, phospho-Syk, Syk, phospho-ERK1/2, ERK1/2, phospho-p38, p38, phospho-JNK, JNK, cytosolic phospholipase A_2_ (cPLA_2_), phospho-cPLA_2_, cyclooxygenase-2 (COX-2), and *β*-actin were purchased from Cell Signaling Technology (Beverly, MA, USA). Specific antibodies against phospho-5-lipoxygenase (5-LO) and 5-LO, and enzyme immunoassay (EIA) kits for prostaglandin E_2_ (PGE_2_), prostaglandin D_2_ (PGD_2_), leukotriene B_4_ (LTB_4_), and leukotriene C_4_ (LTC_4_) were purchased from Cayman Chemical (Ann Arbor, MI, USA). The enzyme-linked immunosorbent assay (ELISA) kits for TNF-*α* and IL-4 were obtained from Bangyi Technologies Inc. (Shanghai, China). Dinitrophenyl- (DNP-) IgE was obtained from Sigma-Aldrich (St Louis, MO, USA), and DNP-bovine serum albumin (BSA) was obtained from Biosearch Technologies Inc. (Novato, CA, USA). All other chemicals were of analytical grade and were purchased from Sigma-Aldrich.

### 2.2. Cell Culture

RBL-2H3 cells were purchased from the Type Culture Collection of the Chinese Academy of Sciences (Shanghai, China). Cells were cultured in DMEM medium supplemented with 10% FBS and antibiotics (100 U/mL penicillin and 100 *μ*g/mL streptomycin) at 37°C in a humidified 5% CO_2_ atmosphere.

### 2.3. Cytotoxicity Assay

Cell respiration served as an indicator of cell viability and was determined by measuring the mitochondrial-dependent reduction of WST-1 to water-soluble tetrazolium salt [19]. Briefly, RBL-2H3 cells were seeded onto a 96-well plate (1 × 10^4^ cells/well) in DMEM with 10% FBS at 37°C overnight. The cells were washed and incubated with DNP-IgE (10 *μ*g/mL) for 24 h. The IgE-sensitized cells were incubated with ME (0–100 *μ*M) for 1 h and stimulated with DNP-BSA (100 ng/mL) for 4 h. WST-1 reagent (10 *μ*L) was added, and the mixture was further incubated for 1 h. Cell viability was determined by measuring the difference in absorbance at a wavelength of 450 nm.

### 2.4. *β*-Hexosaminidase Release Activity

RBL-2H3 cells were incubated in a 24-well plate (2 × 10^5^ cells/well) at 37°C overnight. The cells were washed with 1× PBS and incubated with DNP-IgE (10 *μ*g/mL) for 24 h. The IgE-sensitized cells were incubated with ME (0–100 *μ*M) for 1 h, followed by 4 h incubation with DNP-BSA (100 ng/mL). To measure *β*-hexosaminidase activity, the culture medium was centrifuged (17,000 ×g, 10 min) at 4°C. The supernatant (25 *μ*L) was mixed with 10 mM poly-N-acetyl glucosamine (p-NAG; 50 *μ*L) in 0.1 M sodium citrate buffer (pH 4.5) in a 96-well plate and incubated for 1 h at 37°C. The reaction was terminated by stop buffer (0.1 M Na_2_CO_3_ buffer, pH 10.0). The *β*-hexosaminidase activity was determined by measuring the difference in absorbance at 405 nm. Data were displayed as the mean ± standard deviation (SD) of triplicate experiments.

### 2.5. ELISA

To measure the TNF-*α* and IL-4 concentrations in the culture media, all samples were centrifuged (17,000 ×g, 10 min) at 4°C and stored at −80°C until analysis. The TNF-*α* and IL-4 concentrations were measured using ELISA kits according to the manufacturer's instructions. Data were displayed as the mean ± SD of triplicate experiments.

### 2.6. EIA

To determine the PGE_2_, PGD_2_, LTB_4_, and LTC_4_ concentrations in the culture media, all samples were centrifuged (17,000 ×g for 10 min) at 4°C, and the supernatant was stored at −80°C until analysis. The PGE_2_, PGD_2_, LTB_4_, and LTC_4_ concentrations were measured with EIA kits according to the manufacturer's instructions. Data were displayed as the mean ± SD of triplicate experiments.

### 2.7. Western Blot Analysis

RBL-2H3 cells were seeded onto a 6-well plate (5 × 10^5^ cells/well) in DMEM with 10% FBS at 37°C overnight. The cells were washed and incubated with DNP-IgE (10 *μ*g/mL) for 24 h. The cells were then incubated in ME (0−100 *μ*M) for 1 h and stimulated with DNP-BSA (100 ng/mL) for 4 h. The harvested cells were lysed, and the target protein was resuspended in protein lysis buffer. The cell lysates were separated by sodium dodecyl sulfate-polyacrylamide gel electrophoresis (SDS-PAGE) and transferred to polyvinylidene fluoride (PVDF) membranes. The membranes were then incubated with a 1 : 1,000 dilution of specific antibodies against phospho-Lyn, Lyn, phospho-Syk, Syk, phospho-ERK1/2, ERK1/2, phospho-p38, p38, phospho-JNK, JNK, phospho-cPLA_2_, cPLA_2_, COX-2, and *β*-actin and antibodies against phospho-5-LO and 5-LO. The blots were washed with TBS-T and incubated in a 1 : 5,000 dilution of horseradish peroxidase-conjugated IgG secondary antibodies. The proteins on the membranes were detected using a chemiluminescent reaction, and the membranes were exposed to Hyperfilm ECL. The target protein concentrations were compared to the control concentrations, and the results for each protein were expressed as a density ratio based on a protein standard size marker. The density of each band was determined using ImageJ software.

### 2.8. Statistical Analysis

The results were expressed as mean ± standard deviation (SD) and differences between mean values of normally distributed data were assessed by the one-way analysis of variance (ANOVA) followed by Duncan's test for multiple comparisons. *P* values of 0.05 or 0.01 were considered statistically significant.

## 3. Results

### 3.1. Inhibitory Effect of ME on IgE-Mediated Allergic Response in RBL-2H3 Cells

**Figure 1:**
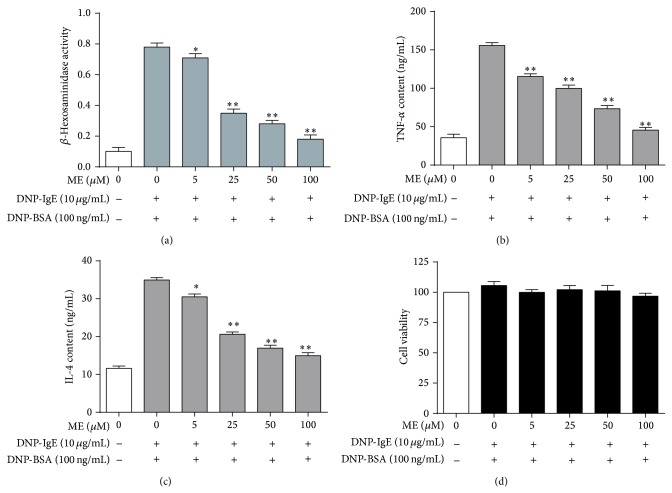
Effect of ME on activity of *β*-hexosaminidase and level of TNF-*α*, IL-4 released in IgE-activated RBL-2H3 cells. RBL-2H3 cells were seeded on a 24-well plate in DMEM with 10% FBS at 37°C overnight, and then the cells were washed and further incubated with DNP-IgE for 24 h. The cells were incubated with ME (0–100 *μ*M) for 1 h and then stimulated by DNP-BSA (100 ng/mL) for 4 h. *β*-Hexosaminidase activity (a) and TNF-*α* level (b) and IL-4 level (c) were determined as described in Section 2. RBL-2H3 cells were seeded on a 96-well plate (2.5 × 10^4^ cells/well) in DMEM with 10% FBS at 37°C overnight, and then the cells were washed and further incubated with DNP-IgE for 24 h. The cells were incubated with ME (0–100 *μ*M) for 1 h, simultaneously treated with DNP-BSA (100 ng/mL) and WST-1 reagent (10 *μ*L), and then incubated for 4 h. Cell viability (d) was determined as described in Section 2. Data represent the mean ± SD of three independent experiments and differences between mean values were assessed by one-way ANOVA. ^*^ *P* < 0.05, ^**^ *P* < 0.01 indicate significant differences compared with the DNP-BSA-treated group.

To determine the optimal concentrations of ME for our study, we assessed the cytotoxicity of ME and antigen (DNP-BSA) cotreatment. We treated the RBL-2H3 mast cells with ME concentrations ranging from 1 to 100 *μ*M in subsequent experiments. The IgE-sensitized RBL-2H3 cells were exposed to ME at various concentrations (0−100 *μ*M) for 1 h and stimulated with 100 ng/mL DNP-BSA for 4 h for the *β*-hexosaminidase assay. ME markedly inhibited the release of *β*-hexosaminidase (Figure 1(a)), which is a general biomarker of degranulation and a hallmark characteristic of allergic reactions caused by allergen exposure. Additionally, the release of TNF-*α* and IL-4, two proinflammatory cytokines, from RBL-2H3 cells was markedly suppressed by ME in a dose-dependent manner (Figures 1(b) and 1(c)). ME treatment (0−100 *μ*M) for 24 h produced no significant cytotoxic effect (Figure 1(d)).

### 3.2. Inhibitory Effects of ME on the Formation of Proinflammatory Lipid Mediators

**Figure 2:**
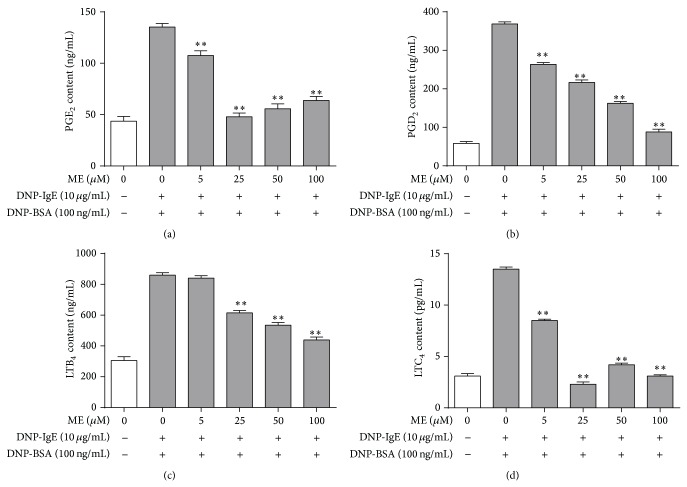
Effect of ME on formation of PGE_**2**_, PGD_2_, LTB_4,_ and LTC_4_ in IgE-activated RBL-2H3 cells. RBL-2H3 cells were seeded on a 24-well plate in DMEM with 10% FBS at 37°C overnight, and then the cells were washed and further incubated with DNP-IgE for 24 h. The cells were incubated with ME (0–100 *μ*M) for 1 h and then stimulated by DNP-BSA for 4 h. The amounts of PGE_2_ (a), PGD_2_ (b), LTB_4_ (c), and LTC_4_ (d) were determined as described in Section 2. Data represent the mean ± SD of three independent experiments and differences between mean values were assessed by one-way ANOVA. ^*^ *P* < 0.05, ^**^ *P* < 0.01 indicate significant differences compared with the DNP-BSA-treated group.

We next examined the effect of ME on the formation of PGE_2_, PGD_2_, LTB_4_, and LTC_4_, which are proinflammatory lipid mediators that regulate allergic response [20–23] produced via arachidonate signaling downstream of IgE-mediated Fc*ε*RI activation [24]. RBL-2H3 cells were preincubated with ME (0–100 *μ*M) prior to antigen challenge, and the formation of PGE_2_, PGD_2_, LTB_4_, and LTC_4_ was measured by EIA assay. As shown in Figure 2, ME markedly inhibited the formation of PGE_2_, PGD_2_, and LTC_4_ and suppressed LTB_4_ formation to a lesser extent. Collectively, these results suggest that ME suppresses allergic inflammation induced by PGE_2_, PGD_2_, LTB_4_, and LTC_4_. This indicates that ME directly inhibits an enzyme involved in prostaglandin and leukotriene biosynthesis.

### 3.3. Regulatory Effects of ME on Enzymes Associated with the Arachidonate Cascade

**Figure 3:**
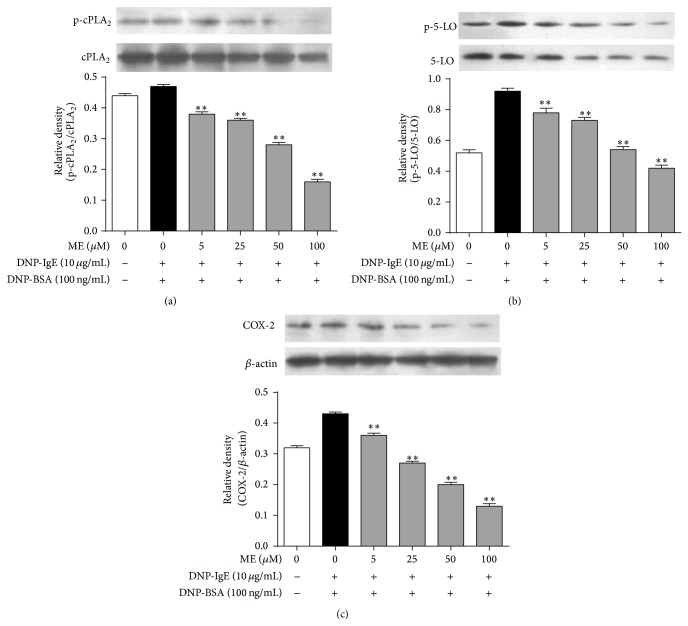
Effect of ME on a late stage of the Fc*ε*RI signal cascade in IgE-activated RBL-2H3 cells. RBL-2H3 cells were seeded on a 6-well plate in DMEM with 10% FBS at 37°C overnight, and then the cells were washed and further incubated with DNP-IgE for 24 h. The cells were incubated with ME (0–100 *μ*M) for 1 h and then stimulated by DNP-BSA for 4 h. The cells were rinsed and lysed with a cell lysis buffer. The expression of p-cPLA_2_, cPLA_2_, p-5-LO, 5-LO, COX-2, and *β*-actin was determined as described in Section 2. Data represent the mean ± SD of three independent experiments and differences between mean values were assessed by one-way ANOVA. ^*^ *P* < 0.05, ^**^ *P* < 0.01 indicate significant differences compared with the DNP-BSA-treated group.

We additionally investigated the antiallergic effects of ME on the activation of enzymes in the arachidonate cascade. Arachidonate cascade activation has been implicated in Fc*ε*RI receptor activation in IgE-activated mast cells [22]. Therefore, we hypothesized that ME, which showed antiallergic effects, would affect cPLA_2_, 5-LO, or COX-2 activation (Figure 3). When the IgE-sensitized RBL-2H3 cells were exposed to ME at various concentrations for 1 h prior to antigen stimulation, phosphorylation of cPLA_2_, the rate-limiting step of the arachidonate cascade, was diminished. Similarly, ME suppressed 5-LO phosphorylation, the rate-limiting step of leukotriene biosynthesis, and inhibited COX-2 expression, which catalyzes the rate-limiting step of prostaglandin biosynthesis. These findings indicate that ME decreases the activation of several targets, including cPLA_2_, 5-LO, and COX-2, suggesting that the antiallergic action of ME may be mediated by arachidonate cascade suppression.

### 3.4. Suppressive Effect of ME on Fc*ε*RI Signaling Pathway

**Figure 4:**
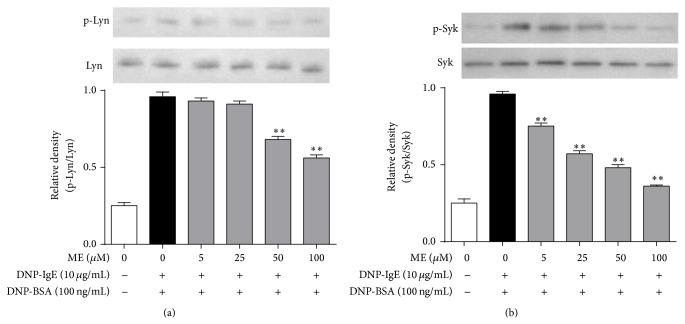
Effect of ME on early stage of Fc*ε*RI cascade in IgE-activated RBL-2H3 cells. IgE-sensitized RBL-2H3 cells were exposed to ME (0–100 *μ*M) for 1 h and then stimulated by DNP-BSA (100 ng/mL) for 10 min. The cells were rinsed with 1× PBS and lysed with cell lysis buffer. The expression of p-Lyn, Lyn, p-Syk, and Syk was determined as described in Section 2. Data represent the mean ± SD of three independent experiments and differences between mean values were assessed by one-way ANOVA. ^*^ *P* < 0.05, ^**^ *P* < 0.01 indicate significant differences compared with the DNP-BSA-treated group.

**Figure 5:**
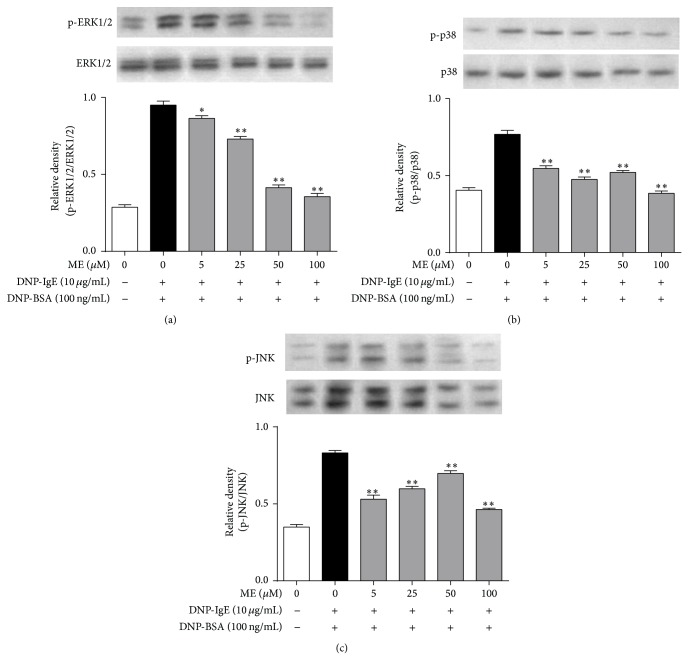
Effect of ME on MAP kinase pathway in IgE-activated RBL-2H3 cells. IgE-sensitized RBL-2H3 cells were exposed to ME (0–100*μ*M) for 1 h and then stimulated by DNP-HSA (100 ng/mL) for 10 min. The cells were rinsed with 1× PBS and lysed with cell lysis buffer. The expression of p-ERK1/2, ERK1/2, p-p38, p38, p-JNK1/2, or JNK1/2 was determined as described in Section 2. Data represent the mean ± SD of three independent experiments and differences between mean values were assessed by one-way ANOVA. ^*^ *P* < 0.05, ^**^ *P* < 0.01 indicate significant differences compared with the DNP-BSA-treated group.

Next, we investigated the mechanism of the antiallergic action of ME. Activation of the Fc*ε*RI receptor induces Lyn and Syk phosphorylation, mediating the degranulation of mast cells [22]. In this respect, ME may affect Lyn or Syk phosphorylation in the early phase of the Fc*ε*RI receptor cascade. When RBL-2H3 cells were preincubated with ME for 1 h before antigen challenge, and the incubation was extended an additional 10 min, the phosphorylation of Syk, but not Lyn, was inhibited in a dose-dependent manner (Figure 4). Notably, ME markedly reduced the expression and phosphorylation of ERK1/2 (Figure 5(a)). Thus, ME could reduce ERK1/2 function by directly suppressing ERK1/2 expression. Additionally, phosphorylation of MAP kinases, such as p38 or JNK, was also suppressed by ME, although p38 phosphorylation was more sensitive to ME (Figures 5(b) and 5(c)).

## 4. Discussion

The essential oil of* Asiasari radix* has many beneficial health effects, exhibiting anti-inflammatory, antibacterial, and antiallergy properties, as well as affecting the respiratory and circulatory systems [25].* Asiasari radix* essential oils contain a considerable number of chemical ingredients, including ME, asarylketone, cineol, safrole, limonene, and eucarvone [26]. Previously, ME was reported to have beneficial effects on inflammation, ischemia, anaphylaxis, and nociception. Our present data demonstrate that ME exerts antiallergic effects in IgE-activated RBL-2H3 cells. ME significantly suppresses degranulation and proinflammatory cytokine release in antigen-sensitized mast cells. Several cytokines play critical roles in allergic inflammation. For example, TNF-*α*, which is secreted from IgE-activated mast cells, plays an important role in allergic responses [27]. Therefore, the inhibitory effect of ME on TNF-*α* formation may indicate its added advantage as an antiallergy agent. During the pathogenesis of allergic disease, IL-4 is crucial for the induction of IgE synthesis and mast cell development [28]. IL-4 also modulates the inflammatory response, owing to its ability to affect adhesion molecule expression and cytokine production in endothelial cells, and promotes growth and activation of neutrophils, mast cells, T cells, and eosinophils [29]. These results suggest that ME significantly inhibits mast cell degranulation and proinflammatory cytokine release.

One possible mechanism of ME-induced antiallergic activity may be its effect on the Fc*ε*RI signal cascade. IgE-induced degranulation in mast cells is associated with activation of the Fc*ε*RI receptor, and this activation induces the release of various inflammatory mediators, including TNF-*α*, leukotrienes, and prostaglandins via phosphorylation of the Lyn/Syk pathway [23]. In turn, the activation of Syk increases intracellular Ca^2+^ and the activation of the MAP kinase family [23]. Thus, Lyn and Syk are important intracellular mediators in early signaling following Fc*ε*RI receptor activation. In the present study, Syk was markedly inhibited by ME, supporting the notion that it is a primary target of ME. In support of this observation, ME significantly reduced the phosphorylation of ERK1/2, p38, and JNK, which are downstream effectors of Fc*ε*RI [23].

In the present study, 100 *μ*M ME obviously inhibited cPLA_2_ and 5-LO phosphorylation and decreased the formation of the 5-LO products, LTB_4_ and LTC_4_. This effect may improve the antiallergy action of ME, because LTB_4_ is a potent chemoattractant and activator of neutrophils and other immune cells in severe asthma [30, 31]. LTC_4_ is a potent spasmogenic agent and an agonist of cysteinyl-LT receptors, which are known to induce chronic inflammatory reactions in allergic diseases [21]. Furthermore, ME also inhibited COX-2 expression and dramatically reduced the levels of the COX-2 products PGE_2_ and PGD_2_, which are enhanced in activated immune cells, including mast cells [20, 32]. The suppressive effects of ME on PGE_2_ formation may contribute to its increased antiallergic activity, as PGE_2_ may mediate asthma development and inflammation associated with IL-4 and IL-5, which are produced by helper T cells [32]. Moreover, the inhibitory effect of ME on PGD_2_ formation may add to the antiallergic action, as PGD_2_ is known to cause bronchoconstriction and vasodilation and increases capillary permeability and mucous production in asthma [20]. Collectively, these findings suggest that ME can reduce allergic reactions through suppression of cPLA_2_ and 5-LO activation and through inhibition of COX-2 activity. Taken together, ME can inhibit allergic reaction by suppressing the activation of Syk, ERK1/2, p38, and JNK and reducing the activity of the enzymes responsible for the biosynthesis of PGD_2_ and LTB_4_. Further, these effects may be extended to anti-inflammatory effects on other cells or tissues. Additionally, the expression of TNF-*α* is associated with p38, JNK, and ERK1/2 activation in the Fc*ε*RI receptor cascade in IgE-activated mast cells [23]. Therefore, the reduction of TNF-*α* formation by ME may provide an additional advantage to ME as an antiallergic agent.

In conclusion, the present study demonstrates that ME has antiallergic effects in IgE-activated RBL-2H3 cells. The mechanisms responsible for its antiallergic effects may involve multiple targets including Sky, ERK1/2, p38, JNK, cPLA_2_, 5-LO, and COX-2. Such effects may provide further information for the application of ME as an antiallergic agent. Therefore, our future studies will focus on providing additional pharmacological evidence to demonstrate this possibility.
